# Supplementary material for: Private investments in climate change adaptation are increasing in Europe, although sectoral differences remain
Source: Commun Earth Environ. 2025 Jun 17;6(1):470. doi: 10.1038/s43247-025-02454-3 (PMC12173939; doi:10.1038/s43247-025-02454-3)
Supplement: Supplementary file 2 — Supplementary Information [file 43247_2025_2454_MOESM2_ESM.pdf]

Private investments in climate change adaptation are increasing in Europe, although sectoral differences remain

Supplementary Information

Ignasi Cortés Arbués[1,*], **Theodoros Chatzivasileiadis**[1], **Servaas Storm**[2], **Olga Ivanova**[3], and **Tatiana Filatova**[1*].

[*]Corresponding authors: i.cortesarbues@tudelft.nl, t.filatova@tudelft.nl

[1]Department of Multi-Actor Systems, Faculty of Technology, Policy and Management, Delft University of Technology, Delft, The Netherlands.

[2]Department of Values, Technology and Innovation, Faculty of Technology, Policy and Management, Delft University of Technology, Delft, The Netherlands.

[3]PBL Netherlands Environmental Assessment Agency, The Hague, The Netherlands.

Contents

Supplementary Note 1: Methods – Description of Hazards Types 2

Supplementary Note 2: Methods – Data Estimation Methodology (provided by kMatrix Data Services) 3

SN.2.1: Brief General Methodology for kMatrix Data Production 3

SN.2.2: Measures, Metrics and Definitions 4

SN.2.3. Data Triangulation – the Cornerstone of kMatrix 5

SN.2.4. Data Confidence Levels 7

SN.2.5. Difference Between Data Confidence Levels and Data Confidence Intervals 7

SN.2.6. "Deep Dive" Example – Sustainable drainage and water management in the Netherlands in 2018 7

SN.2.7. Full Taxonomy of Adaptation Activities (per sector and hazard type) 13

Supplementary Note 3: Results – Growth Rates in Adaptation Spending 22

Supplementary Note 4: Methods – Regression Sensitivity Analysis 24

Supplementary References 26

Supplementary Note 1: Methods – Description of Hazards Types

Below we describe what kind of events are included within the 5 hazard type categories in the paper, as provided by the adaptation spending data provider, kMatrix Ltd[1]. Note that the data underlying the analysis in the paper is the result of summing all individual investments to adapt to a specific hazard type associated to a specific country-sector combination per year (e.g., the sum of all Flooding investments by the Manufacturing sector in Austria in 2019 result in one data point). These definitions and examples were provided by Sarah Howard at kMatrix Data Services and have been edited by I.C.A for conciseness.

Supplementary Table 1*:* Description of the aggregation of climate hazard categories associated to public and private adaptation investments included in the study.

Hazard Type	Description
**Flooding**	The three main types of flooding events are aggregated, namely:<br>• Fluvial Flooding<br>• Pluvial Flooding<br>• Coastal Storm Surges
**Heatwave**	A heatwave is defined as 3 days of hot weather, exceeding a given localized threshold, based on the expected conditions of the area at that time of year. Some considerations in the quantification of heatwave-related investments are the following:<br>• The dataset does not track heatwaves per se, rather it tracks purchasing patterns and reactions to reported weather conditions.<br>• Only multi-reactions are counted, i.e. a purchase of a single air conditioning unit would not be counted, whereas a large number of air conditioning units being purchased would be counted, but only in instances where it is associated with a heatwave.
**Drought**	A drought is defined as a prolonged period of abnormally low rainfall, leading to shortage of water; it also includes anthropogenic drought through re-direction of water courses.
**Wildfires**	A wildfire is defined as an unplanned, unwanted, uncontrolled fire, burning in a natural area such as grassland, forest, scrub, etc. Both events started naturally (e.g. lightning strike) and artificially (i.e., by human beings) are included, as wildfires can be worsened by climatic conditions.
**Other**	A mixed classification of minor hazards, including:<br>• **Windstorms**: strong winds able to cause at least mild damage to trees and buildings; may be accompanied by precipitation, but only wind-related damage is included here.<br>• **Landslides**: movement of a mass of rock, debris or earth down a slope. Only weather-related events are included, (i.e., landslides caused by coastal erosion, mining activity, earthquakes, etc.).<br>• **Sinkholes**: cavities in the ground caused by water erosion, especially in limestone formation. They usually appear unexpectedly, can be significant in depth and cause catastrophic damage. Those associated with mine shafts are not included in the dataset.

Supplementary Note 2: Methods – Data Estimation Methodology (provided by kMatrix Data Services)

This section presents the methodology through which our external data provider (kMatrix Data Services) has built the adaptation expenditure dataset underlying this study. It includes a brief description of the general methodology, an example of a sector-hazard taxonomy of measures, an explanation of their data triangulation approach and considerations made in the inclusion of their sources (i.e., data confidence levels), and finally a worked example of how a datapoint is constructed using the methodology (e.g.. Flooding Adaptation for the agricultural sector in The Netherlands in 2018). After the worked example, we provide extensive tables mapping adaptation measures to NACE 2 sectors and to hazard types. This section has been drafted by kMatrix Data Services and edited minimally by I.C.A. for the sake of readability within this Supplementary Information. Further questions on the methodology and data possibilities can be directed to enquiries@kmatrix.org.

The authors would like to explicitly thank Sarah Howard at kMatrix Data Services for the general support and communication regarding the data management, and for writing this extensive explanation of their methodology, including a worked example.

SN.2.1: Brief General Methodology for kMatrix Data Production

This methodology refers to the data triangulation process used by kMatrix Data Services as commissioned by TU Delft within the scope of the ERC Project SCALAR (Grant No. 758014). It includes a description of the methodology and then a 'deep dive' on a single datapoint.

kMatrix uses a unique data triangulation methodology, developed with Professor R. Jaikumar of Harvard University over 35 years ago[2]. The process was originally developed to look at individual companies, providing evidenced data for development. As such, sectors are classified from the 'bottom up,' collecting activities from the most finite level of granulation and grouping them into successive levels of detail. This diagram provides an example of the depth of detail possible, using the Low Carbon Environmental Goods and Services (LCEGS) sector as an example:

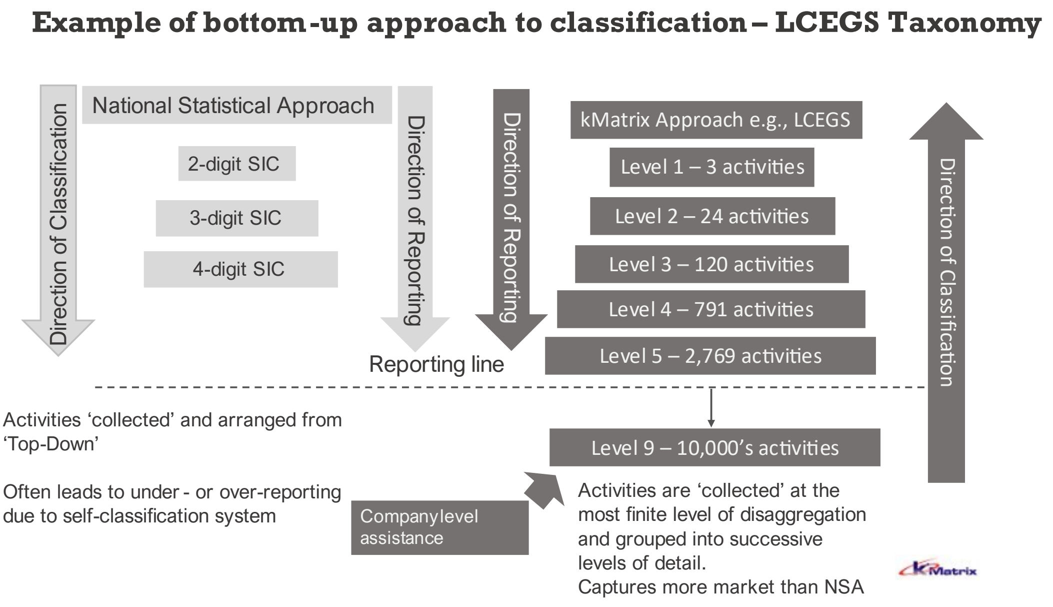

Supplementary Figure 1: Visualisation of the bottom-up used by kMatrix Data Services to provide industry data at different levels of regional and sectoral aggregation.

This is quite different to the National Statistical Approach, which classifies from the 'top down,' with a company choosing their 2-digit code, then successive codes down through the classification system. The SIC system is very good as a national accounting system, but it struggles with hard to measure sectors such LCEGS or Adaptation and Resilience. Here, the kMatrix system of data collection, which triangulates transactional data from many thousands of sources, provides the flexibility of a definition tailored to the sector being studied. Although the sector is classified from the bottom up, the sector taxonomy is reported from the sector level down, through a series of levels of complexity.

This process has measured the LCEGS sector for the Greater London Authority and the UK for over a decade. kMatrix also collaborate with academic colleagues in several fields, co-authoring academic papers, which are peer-reviewed and published in academic journals including Nature, Climate Services, and the Lancet. kMatrix is also published in the House of Lords for the Security sector and provide the European Commission with Cybersecurity data across Europe. Example sectors the process has been applied to, where evidence is available in the public domain via clients publishing reports or published peer-reviewed academic journals include:

- Domestic Energy Retrofit in the North East https://evidencehub.northeastlep.co.uk/domestic-retrofit-skills-needs-assessment
- Low carbon environmental goods and services sector: https://www.london.gov.uk/sites/default/files/london_low_carbon_market_snapshot_-_2019.pdf and https://www.sustainabilitywestmidlands.org.uk/resources/midlands-low-carbon-sector-study/
- The green Economy: https://rgs-ibg.onlinelibrary.wiley.com/doi/pdf/10.1002/geo2.36 and https://www.nature.com/articles/s41599-019-0329-3
- Adaptation economy: https://www.nature.com/articles/nclimate2944
- Carbon Finance: https://www.nature.com/articles/nclimate1492?draft=marketing
- Weather and Climate: https://advances.sciencemag.org/content/3/5/e1602632.full
- Climate Services: https://www.sciencedirect.com/science/article/pii/S2405880719300494?via%3Dihub

SN.2.2: Measures, Metrics and Definitions

Metrics have been applied to a taxonomy of adaptation measures and then reported by Sector and Hazard (adaptation measures by NACE 2 code are in **Supplementary Table 4**; adaptation by hazard are in **Supplementary Table 5**; and a section of the taxonomy in **Supplementary Table 6**). Flooding within Agriculture has been used as a deep dive example:

Supplementary Table 2: Adaptation Measures for the Flooding hazard type for the sector A. Agriculture, Forestry and Fishing.

NACE Code	Hazard	Adaptation Measure
A. Agriculture, Forestry and Fishing	Flooding	Adaptation and Management of Farm Equipment (incl. training)
		Advanced risk modelling
		Advanced water management technologies
		Consulting
		Cultivation of New Crops Enabled by Climate Change
		Erection of flood barriers
		Evacuation services
		Excavation of new or extension of existing dykes/ditches/droves

		Finance
		Forestry Services (incl. Afforestation)
		Green roofing and walls
		Improved New Species
		Improvements in Agricultural Management (incl. crop & soil and training)
		Improvements in Forest Management
		Installation of green infrastructure e.g., reed beds, river re-wiggling etc.
		Installation of porous hard standing
		Installation of temporary flood barriers
		Insurance
		Pumping equipment
		Retrofit building services
		Sand bags
		Stormwater mitigation measures
		Sustainable drainage and water management
	Heatwave	Relevant Activities Included
	Drought	Relevant Activities Included
	Wildfires	Relevant Activities Included
	Other	Relevant Activities Included
Other NACE Codes	Hazards Repeated	Relevant Activities Included

SN.2.3. Data Triangulation – the Cornerstone of kMatrix

kMatrix uses a propriety data triangulation methodology to calculate over 100 metrics for many sectors including Domestic Retrofit, Space, Climate Services, Green Economy, Marine, Security, Cybersecurity, Adaptation & Resilience, Water, Design and others.

The same general research methodology is used across all sectors and metrics, while the requirements for each industrial sector research project vary, the methodology and process always follow the same five key stages:

1. Define – Identify, select and group the target market activities, whilst conducting an initial check for the right volume and quality of data sources
2. Assemble – Populate the data measures for each market activity, carefully filtering the core data sources to ensure that confidence levels are within bounds
3. Check – Apply quality assurance checks to ensure data accuracy/consistency across market activities and different countries and, in some cases, cross-check with different sector values
4. Validate – Sense-check and spot-check market data values against specific projects/authoritative sources/expert knowledge/customers or clients

5. Publish – Recalculate, update confidence levels and publish research data set

This process is illustrated in more detail by the Sector Research Model (**Supplementary Figure 2**), where the numbers correspond to the process stages above.

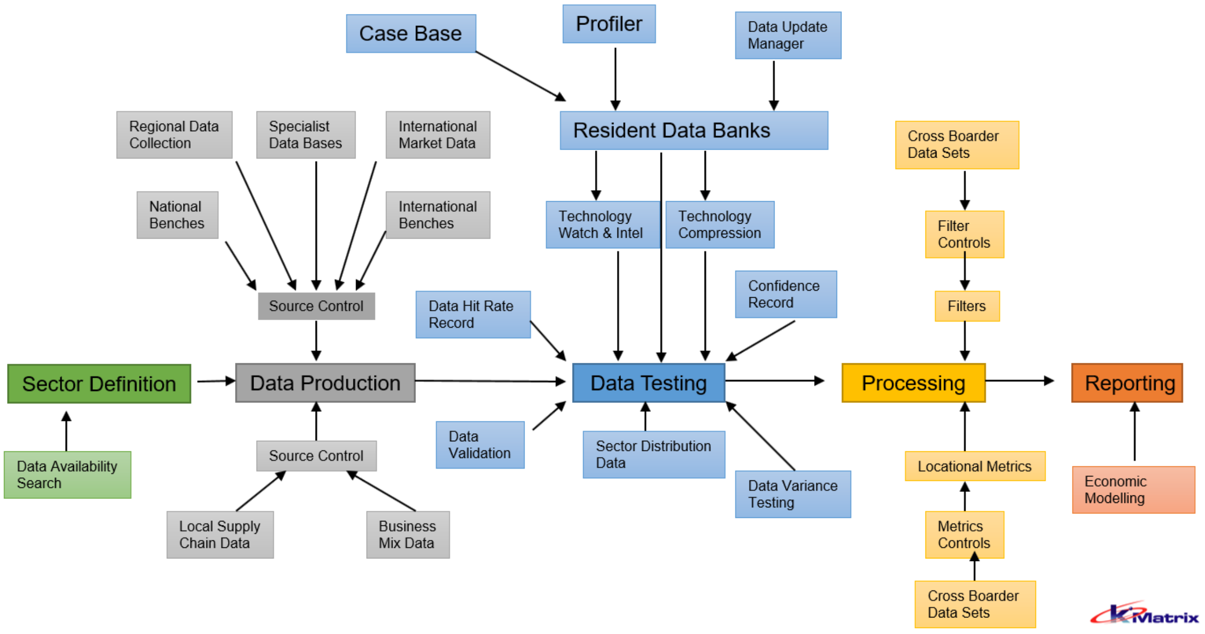

Supplementary Figure 2: Flowchart representation of the Sector Research Model used by kMatrix Data Services. Each colour represents one of the steps presented above: 1. Define (Green); 2. Assemble (Grey); 3. Check (Blue); 4. Validate (Yellow); 5. Publish (Orange).

The research model used by kMatrix comprises distinct but iterative phases. This is because kMatrix needs to "create" its own data before it can analyse it. This is fundamentally different from any SIC-based sector analysis that takes its data as a "given."

Within the above model a range of different research methods are employed. Some aspects of the research system are semi-automated (necessary when dealing with large volumes of data), but the rulesets and software algorithms are based upon 35+ years' experience in compiling and reporting complex markets and all final decisions about reported outputs are subject to intense analyst scrutiny.

The research methods employed include:

- Desk research to define sector content and determine sector boundaries
- Industrial templates that identify core and supply chain activities in detail for inclusion in sector definition
- Data discovery tools to identify new data and sources relating to the defined sector activities
- Data coding systems to ensure that sector, company-level data and other classification systems are aligned for analysis and reporting purposes
- Data management techniques and systems to maintain existing source libraries and integrate them with new source materials
- Software systems with defined (but flexible) rulesets to filter source content
- Semi-automated processes for modelling and calculating data values from selected source lists
- Knowledge base of case study materials that can be accessed to help fill data gaps and provide industrial performance benchmarks
- Quality assurance processes and tools that check all values against a range of international, national and industrial comparators
- Data management and visualisation tools for presenting and reporting data

SN.2.4. Data Confidence Levels

All kMatrix datasets incorporate a measure of data Confidence Level. This is partly a mathematical function of the historic hit-rate of sources in terms of their previous forecasting accuracy for each metric and activity being measured. Confidence levels vary by activity, measure, geography and forecast year. Typically a confidence level of above 90% is achievable. The focus in the research process is to obtain an across-the board level of data confidence rather than attempt to achieve 100% confidence in any one set of numbers. As each data point has its own set of sources, so each data point has its own level of data confidence. Data confidence levels can vary according to how mature or emergent a product or service is, and how mature the reporting systems may be or for any given country. Developed nations typically have a wider range of robust sources to choose from.

kMatrix tracks the accuracy of sources over a period of time, with Confidence Levels being built up from the history of the accuracy of a source for a specific metric. The history or 'hit-rate' of a source is held for each metric, with some sources accepted for some metrics and rejected for other metrics, dependent on how accurate they have been in the past. Hit-rates are 'live' and the hit-rate of a source can change over time, as their performance changes over time, with some sources hit-rates being tracked over the last 35 years. The ability to select, reject and assess the validity of the extensive source list, for different markets, metrics and geographies is a fundamental aspect of the kMatrix process.

SN.2.5. Difference Between Data Confidence Levels and Data Confidence Intervals

Data Confidence Intervals are often used within survey data, where they give an indication of the degree of uncertainty of an estimate within a sample, specifying the range of values likely to contain the unknown population value, by defining upper and lower limits within a data sample.

The difference between this and the Confidence Level above, is it provides the variance, but it weights all data as being equally valid and does not factor in the likely validity of each data source.

In summary, Data Confidence Levels are different to Data Confidence Intervals:

- ***Data Confidence Levels*** estimate the accuracy of a triangulated data point, based on the likely accuracy of each of the multiple sources of data that are used in the triangulation process, through tracking historical accuracy
- ***Data Confidence Intervals*** provide a range of values where the true number could lie, but do not provide detail on the validity of the values used within the production of the range

Confidence Intervals are not provided within the dataset because the data sources within the triangulation process for each datapoint are varied, created for many different reasons and will each have an individual hit-rate.

SN.2.6. "Deep Dive" Example – Sustainable drainage and water management in the Netherlands in 2018

This example provides an illustration of how the multi-source approach has been used to calculate the values within the study. The reported value for Flooding in the Netherlands in 2018 was €25.18m. As illustrated in **Supplementary Table 1**, there are twenty-three adaptation measures in agricultural flooding, of which, sustainable drainage and water management is one. This deep dive illustrates the

process of triangulating the value for this activity. The activity values are then aggregated to produce the value for the hazard within the A. Agriculture, Forestry and Fishing NACE 2 Code.

The multi-source approach includes six stages:

1. Select the data point
2. Identify the source data
3. Select sources for further analysis
4. Triage the sources to achieve a more consistent range of values
5. Calculate the mean value from the sources
6. Calculate the confidence level

Stage 1 involves selecting the data point. In this example, the data point is Netherlands purchase of sustainable drainage and water management in 2018.

Stage 2 involves identifying the data sources that are relevant to the calculation of the data point and is the source list that is used to calculate the value, for this datapoint, it includes 3,802 sources.

Stage 3 involves an initial sort and selection from the full list of sources to identify those of the most direct relevance to the data point. They are rejected if they are duplicates, i.e. using 3rd party data, if their hit rate is too low or if the value is excessively high or low with no evidence for deviation.

Stage 4 of the process involves "smoothing" the results by excluding the outlier values from the final calculation. In this case, 98 sources were ultimately selected and these are shown in **Supplementary Table 3** and are labelled Source 1 through to Source 98. Their selection depends upon several factors stored within our source management system (columns 6-9 of **Supplementary Table 3**). These are:

- Value reported – only sources showing value that are proximate to other values are included
- Year of data – ideally sources should be current, in this case all are 2018
- Hit rate over the past 5 years – level of assessed accuracy for this source over 5 years
- Number of times accessed – number of times this source has been used previously for this purpose i.e. same data point different years, identical data point different country etc.
- Triangulated – is the data extracted from a larger data set for the purposes of comparison?

Supplementary Table 3 shows that the 98 sources were all current, with purchase values between €8.66m and €10.50m, hit rates of between 76.3% and 95.9% and had been accessed previously between 81 and 158 times each. The source list is split (61/37) between triangulated sources and those that are not.

All the values in the data set are unique, which means that multiple sources that may quote the same value (possibly from an identical source) are eliminated from the final selection.

Supplementary Table 3: Long list of sources associated to the measure of Sustainable Drainage and Water Management in the sector A. Agriculture, Forestry and Fishing in the Netherlands in 2018. Some data sources are confidential, and are thus labelled with a number in the "DATA Source" column instead of the source's name.

No	DATA Source	Purchase Value €m	Country	Year of Data	Hit Rate History %	Times Accessed	Triangulated	Accept/ Reject
1	Agricultural Engineers Association AEA	10.49	Netherlands	2018	90.2	120	YES	accept
2	Arboricultural Association	8.71	Netherlands	2018	82.9	121	YES	accept
3	Association of Consulting Engineers	9.40	Netherlands	2018	88.7	119	NO	accept
4	Atmospheric Research and Information Centre	9.74	Netherlands	2018	88.3	93	YES	accept

5	Australasian Institute of Marine Surveyors	10.50	Netherlands	2018	83.8	99	YES	accept
6	Australian Marine Sciences Association	9.80	Netherlands	2018	87.0	96	NO	accept
7	Bell flow Systems	9.23	Netherlands	2018	77.5	148	YES	accept
8	BNP	8.81	Netherlands	2018	95.0	154	NO	accept
9	Both ENDS environment and development service	8.86	Netherlands	2018	87.1	102	YES	accept
10	Chartered Institution of Water and Environmental Management	9.93	Netherlands	2018	88.5	114	NO	accept
11	Chartered Institution of Water and Environmental Management (CIWEM)	10.18	Netherlands	2018	87.3	154	YES	accept
12	Civil engineering contractors Association	10.46	Netherlands	2018	85.0	158	YES	accept
13	Climate Action Network - Europe (CAN-Europe)	8.95	Netherlands	2018	90.6	85	YES	accept
14	Confederation of Construction Specialists	9.90	Netherlands	2018	93.2	149	NO	accept
15	County Clean Environmental	10.26	Netherlands	2018	78.8	92	YES	accept
16	Emergingpe	9.68	Netherlands	2018	84.6	143	YES	accept
17	EMP Global LLC	9.81	Netherlands	2018	76.3	116	NO	accept
18	EPP Europe	9.53	Netherlands	2018	95.9	106	YES	accept
19	ESRC Data Archive	8.75	Netherlands	2018	76.9	158	NO	accept
20	Fauna and Flora International	8.94	Netherlands	2018	93.1	150	NO	accept
21	Federation for the Repair & Protection of Structures	8.76	Netherlands	2018	94.4	83	NO	accept
22	Forest Stewardship Council	8.98	Netherlands	2018	82.8	89	YES	accept
23	Forests and the European Union Resource Network (FERN)	9.21	Netherlands	2018	90.7	84	NO	accept
24	Forests and the European Union Resource Network (FERN)	9.90	Netherlands	2018	90.9	139	YES	accept
25	Friends of Nature	9.46	Netherlands	2018	88.4	99	YES	accept
26	Global Financial Data	10.38	Netherlands	2018	78.2	95	YES	accept
27	Gravity Financial, LLC	9.55	Netherlands	2018	86.5	107	NO	accept
28	Hot Water Association (HWA)	10.03	Netherlands	2018	79.3	130	YES	accept
29	ICCO	9.56	Netherlands	2018	84.0	146	YES	accept
30	IMCA - International Marine Contractors Association	9.06	Netherlands	2018	94.8	102	NO	accept
31	IMPA International Marine Purchasing Association	10.02	Netherlands	2018	81.0	154	YES	accept
32	Institute of Civil Engineers	9.56	Netherlands	2018	86.6	85	NO	accept
33	Institute of Highway Engineers	9.26	Netherlands	2018	93.1	153	YES	accept
34	Institute Of Marine Engineers	9.66	Netherlands	2018	93.4	86	NO	accept
35	Institution of Structural Engineers	8.67	Netherlands	2018	78.5	145	YES	accept

36	International Association of Dredging Companies	9.25	Netherlands	2018	90.7	135	NO	accept
37	Leonard Green & Partners	10.26	Netherlands	2018	90.7	86	NO	accept
38	LeverPoint Management, LLC	9.87	Netherlands	2018	89.5	89	YES	accept
39	Milieudefensie - Friends of the Earth Netherlands	8.82	Netherlands	2018	84.1	105	YES	accept
40	Society For Underwater Technology	9.91	Netherlands	2018	88.8	83	YES	accept
41	1769	9.81	Netherlands	2018	88.2	154	YES	accept
42	2014	10.31	Netherlands	2018	92.0	145	NO	accept
43	2267	9.70	Netherlands	2018	95.9	117	NO	accept
44	2653	10.15	Netherlands	2018	89.7	144	NO	accept
45	4496	9.01	Netherlands	2018	86.5	121	YES	accept
46	5262	9.24	Netherlands	2018	93.5	82	YES	accept
47	6841	10.06	Netherlands	2018	94.8	88	YES	accept
48	7624	10.28	Netherlands	2018	79.2	154	YES	accept
49	9416	10.46	Netherlands	2018	82.9	122	YES	accept
50	10289	9.38	Netherlands	2018	85.4	114	NO	accept
51	10367	9.50	Netherlands	2018	94.1	139	YES	accept
52	11100	9.85	Netherlands	2018	85.9	91	NO	accept
53	11487	9.64	Netherlands	2018	82.8	154	YES	accept
54	12261	10.26	Netherlands	2018	81.9	128	YES	accept
55	12296	9.97	Netherlands	2018	86.0	139	YES	accept
56	13020	9.28	Netherlands	2018	87.6	106	NO	accept
57	14756	8.83	Netherlands	2018	79.3	144	YES	accept
58	16869	8.74	Netherlands	2018	88.6	100	NO	accept
59	17370	8.88	Netherlands	2018	83.4	143	YES	accept
60	17483	10.40	Netherlands	2018	83.5	158	YES	accept
61	17949	10.23	Netherlands	2018	85.0	147	YES	accept
62	18185	8.83	Netherlands	2018	87.7	131	NO	accept
63	19983	10.41	Netherlands	2018	79.0	142	YES	accept
64	21217	9.19	Netherlands	2018	79.3	151	YES	accept
65	22589	9.72	Netherlands	2018	79.8	88	YES	accept
66	22719	9.40	Netherlands	2018	89.3	81	NO	accept
67	23269	9.32	Netherlands	2018	79.9	83	YES	accept
68	23790	9.29	Netherlands	2018	93.8	151	NO	accept
69	24259	10.02	Netherlands	2018	94.0	104	YES	accept
70	25585	10.35	Netherlands	2018	90.9	88	YES	accept
71	25750	9.22	Netherlands	2018	85.0	117	NO	accept

72	25804	9.63	Netherlands	2018	76.3	113	YES	accept
73	27317	9.60	Netherlands	2018	82.5	86	NO	accept
74	28324	8.93	Netherlands	2018	78.4	88	YES	accept
75	29200	9.78	Netherlands	2018	83.8	103	YES	accept
76	31292	9.60	Netherlands	2018	81.9	136	YES	accept
77	32922	9.51	Netherlands	2018	93.4	100	NO	accept
78	33574	9.71	Netherlands	2018	91.9	90	NO	accept
79	34048	10.08	Netherlands	2018	79.4	156	YES	accept
80	36086	9.81	Netherlands	2018	91.2	84	YES	accept
81	36167	9.10	Netherlands	2018	94.0	154	YES	accept
82	38043	10.05	Netherlands	2018	82.0	111	YES	accept
83	38730	9.76	Netherlands	2018	90.3	141	NO	accept
84	41009	10.03	Netherlands	2018	82.4	138	NO	accept
85	41667	10.35	Netherlands	2018	83.7	108	NO	accept
86	50400	8.72	Netherlands	2018	80.3	107	YES	accept
87	50690	8.83	Netherlands	2018	80.3	140	NO	accept
88	52717	9.81	Netherlands	2018	90.7	138	YES	accept
89	53423	9.83	Netherlands	2018	79.5	125	YES	accept
90	53889	8.83	Netherlands	2018	93.3	137	YES	accept
91	54622	9.05	Netherlands	2018	81.9	143	NO	accept
92	55998	8.69	Netherlands	2018	93.3	94	YES	accept
93	56638	8.95	Netherlands	2018	92.3	144	YES	accept
94	56788	10.24	Netherlands	2018	89.9	88	NO	accept
95	59627	9.31	Netherlands	2018	91.3	140	YES	accept
96	60091	9.82	Netherlands	2018	81.6	101	NO	accept
97	60494	10.06	Netherlands	2018	89.7	104	YES	accept
98	69476	8.66	Netherlands	2018	94.6	103	YES	accept

Typically, the process for deciding which sources to include/exclude as part of the final calculation is visualised using radar charts. These charts are a key feature of the QA system and provide a rapid insight into the variation in source values. A radar showing sharp and frequent spikes is due for radical surgery, while a radar with a more consistent pattern (representing a degree of consensus in the sources) is subject to judicious pruning. An example of an outlier would be a good which is usually £10, but someone is selling for £90 on Amazon, this would be considered an outlier and removed from the dataset. These are removed from the dataset because an overpriced good can distort a dataset. This process is undertaken by an analyst (not an algorithm) and can take several iterations before a satisfactory final selection of sources is achieved. The final radar chart based upon the final selection of sources is shown in **Supplementary Figure 3**.

Stage 5 involves the calculation of the mean value from the final list of 98 sources, now ranging between €8.66m and €10.50m. The adjusted mean value from this range is €9.58m.

Stage 6 calculates the confidence level for the data point, which is an indication of the validity, based on the historic hit-rate of the sources used to triangulate the data point, in this case the confidence level is 92%.

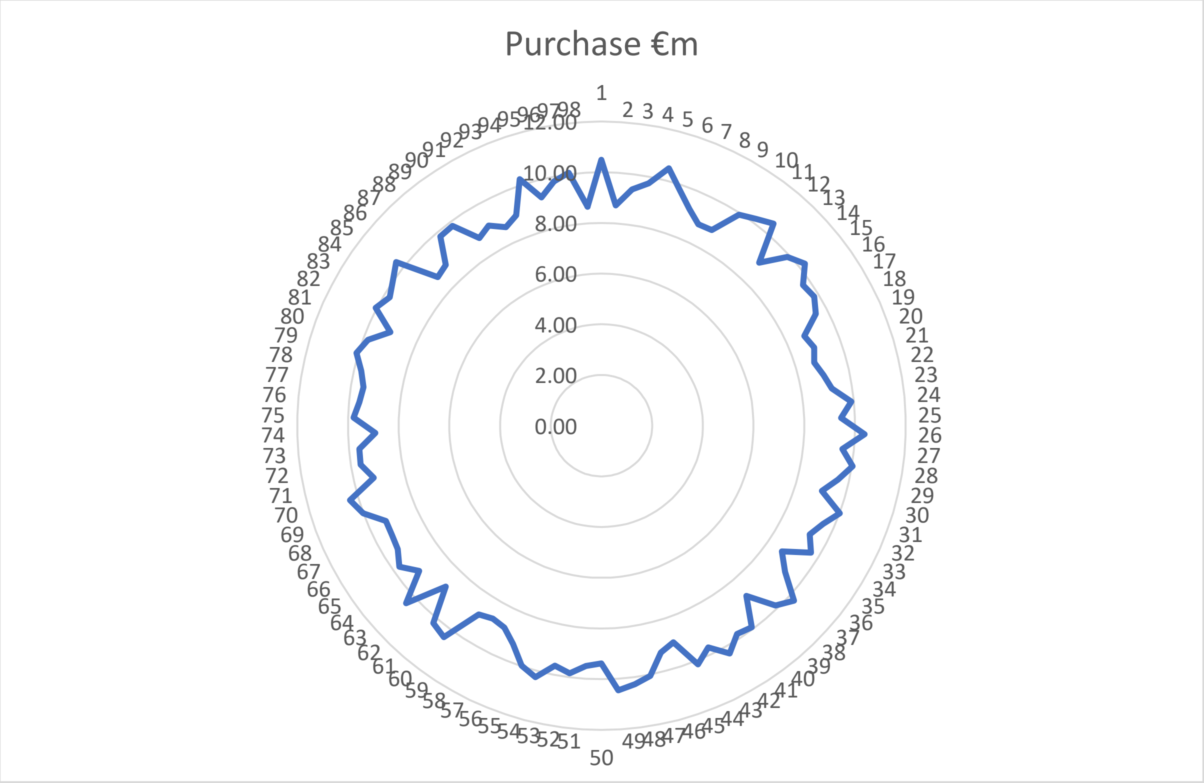

Supplementary Figure 3: Radar Chart of estimated expenditure of the final 98 sources in the example "Sustainable drainage and water management" in Agriculture in the Netherlands in 2018.

Each data point that progresses through this process is then subject to further checks that ensure that the data point value is consistent with:

- Values in previous year's data
- Comparative values of Purchase compared with Purchase levels of same activity in other countries
- Comparative analysis of Purchase values when related to other Installation services in the same category or family of services
- Consistency in purchase trends over time
- Consistency in confidence levels over time

Where any uncertainty about the final data point remains, this may be due to either market uncertainty or data uncertainty. In the case of market uncertainty, the value remains unchanged, but in the case of data uncertainty, then Stages 1-6 are repeated, but this time with additional or alternative data sources.

This datapoint is then aggregated with the other activities within the Agricultural Flooding for the Netherlands for 2018, giving a reported value of €25.18m.

SN.2.7. Full Taxonomy of Adaptation Activities (per sector and hazard type)

Supplementary Table 4: Adaptation Measures Mapped to NACE 2 Rev Code. Note that some activities such as Tree surgery and vegetation management are not present in Agriculture because they are generally performed in-house. Similarly, water use reduction technologies and wastewater management are also not present in Agriculture as they are incorporated in adaptation of farm equipment, improved agricultural management and green infrastructure.

Adaptation Measure	A. Agriculture, Forestry and Fishing	B. Mining and Quarrying	C. Manufacturing	D. Electricity, Gas, Steam and Air Conditioning Supply	E. Water Supply; Sewerage, Waste Management and Remediation	F. Construction	G. Wholesale and Retail Trade; Repair of Motor Vehicles and	H. Transportation and Storage	I. Accommodation and Food Service Activities	J. Information and Communication	K. Financial and Insurance Activities	L. Real Estate Activities	M. Professional, Scientific and Technical Activities	N. Administrative and Support Service Activities	O. Public administration and defence; compulsory social security	P. Education	Q. Human Health and Social Work Activities	R. Arts, Entertainment and Recreation	S. Other Service Activities
Adaptation and Management of Farm Equipment (incl. training)	•																		
Advanced risk modelling	•	•	•	•	•	•	•	•	•	•	•	•	•	•	•	•	•	•	•
Advanced water management technologies	•	•	•	•	•	•	•	•	•	•	•	•	•	•	•	•	•	•	•
Aerial wildfire extinguishing equipment															•				
Consulting	•	•	•	•	•	•	•	•	•	•	•	•	•	•	•	•	•	•	•
Cultivation of New Crops Enabled by Climate Change	•																		
Development of Drought-Resistant Seeds	•																		
Earth observation services															•				
Embedding Climate Change Risk in Energy Infrastructure Planning				•															

Energy efficient cooling of buildings	•	•	•	•	•	•	•	•	•	•	•	•	•	•	•	•	•	•	•
Erection of flood barriers	•	•	•	•	•	•	•	•	•	•	•	•	•	•	•	•	•	•	•
Evacuation services	•	•	•	•	•	•	•	•	•	•	•	•	•	•	•	•	•	•	•
Excavation of new or extension of existing dykes/ditches/droves	•					•		•							•				
Finance	•	•	•	•	•	•	•	•	•	•	•	•	•	•	•	•	•	•	•
Firebreak and firefighting equipment (where related to wildfires)															•				
Firebreak operations															•				
Flood Protection for Power Stations etc				•															
Forestry Services (incl. Afforestation)	•																		
Gabion installation		•	•	•	•	•	•	•	•	•	•	•	•	•	•	•	•	•	•
General supercooling equipment (multi-sector, includes vehicles, manufacturing equipment etc.)	•	•	•	•	•	•	•	•	•	•	•	•	•	•	•	•	•	•	•
Green roofing and walls	•	•	•	•	•	•	•	•	•	•	•	•	•	•	•	•	•	•	•
Heat-resistant infrastructure (e.g., roads)	•	•	•	•	•	•	•	•	•	•	•	•	•	•	•	•	•	•	•
Hydropower Reservoir Stations: increase in dam height				•															
Improved New Species	•																		
Improvements in Agricultural Management (incl. crop & soil and training)	•																		
Improvements in Forest Management	•																		

Increase Robustness of Transmission Grids				•															
Installation of air management systems	•	•	•	•	•	•	•	•	•	•	•	•	•	•	•	•	•	•	•
Installation of green infrastructure e.g., reed beds, river re-wiggling etc.	•														•				
Installation of porous hard standing	•	•	•	•	•	•	•	•	•	•	•	•	•	•	•	•	•	•	•
Installation of temporary flood barriers	•	•	•	•	•	•	•	•	•	•	•	•	•	•	•	•	•	•	•
Installation of thermal and UV reflective surfaces to buildings	•	•	•	•	•	•	•	•	•	•	•	•	•	•	•	•	•	•	•
Insurance	•	•	•	•	•	•	•	•	•	•	•	•	•	•	•	•	•	•	•
Management of External Mineral and Energy Resources			•																
Management of Timber Resources			•																
New Distribution Systems				•															
New Transmission Systems				•															
Pest Suppression Systems and Practices	•																		
Pumping equipment	•	•	•	•	•	•	•	•	•	•	•	•	•	•	•	•	•	•	•
Retrofit building services	•	•	•	•	•	•	•	•	•	•	•	•	•	•	•	•	•	•	•
Sand bags	•	•	•	•	•	•	•	•	•	•	•	•	•	•	•	•	•	•	•
Sinkhole engineering services		•	•	•	•	•	•	•	•	•	•	•	•	•	•	•	•	•	•
Smokejumper															•				
Solar cooling (PV powered) to cope with energy demand peaks (only when associated with heatwaves)				•															

Specialist insulative installation	•	•	•	•	•	•	•	•	•	•	•	•	•	•	•	•	•	•	•
Storm Water (management, treatment, planning)				•	•														
Stormwater civil engineering for new developments				•	•	•									•				
Stormwater mitigation measures	•	•	•	•	•	•	•	•	•	•	•	•	•	•	•	•	•	•	•
Structural Surveys	•	•	•	•	•	•	•	•	•	•	•	•	•	•	•	•	•	•	•
Supply Chain Management (all)							•												
Supply Chain Management (not mineral or timber)			•																
Sustainable drainage and water management	•	•	•	•	•	•	•	•	•	•	•	•	•	•	•	•	•	•	•
Sustainable Sourcing Consulting							•												
Training (Wildfire)															•				
Tree surgery where storm-related		•	•	•	•	•	•	•	•	•	•	•	•	•	•	•	•	•	•
Underground Cable Installation				•															
Underpinning services for landslide mitigation																			•
Vegetation management for land stabilization		•	•	•	•	•	•	•	•	•	•	•	•	•	•	•	•	•	•
Wastewater management and processing of sewage (drought-relevant activities only)		•	•	•	•	•	•	•	•	•	•	•	•	•	•	•	•	•	•
Water Irrigation	•																		
Water use reduction technologies		•	•	•	•	•	•	•	•	•	•	•	•	•	•	•	•	•	•

Supplementary Table 5: Adaptation Measures Mapped to Hazard Type.

Adaptation Measure	Flooding	Heatwave	Drought	Wildfire	Other
Adaptation and Management of Farm Equipment (incl. training)	•	•	•	•	
Advanced risk modelling	•	•	•	•	•
Advanced water management technologies			•		
Aerial wildfire extinguishing equipment				•	
Consulting	•	•	•	•	•
Cultivation of New Crops Enabled by Climate Change	•	•	•	•	
Development of Drought-Resistant Seeds			•		
Earth observation services				•	
Embedding Climate Change Risk in Energy Infrastructure Planning	•	•	•	•	•
Energy efficient cooling of buildings		•			
Erection of flood barriers	•				
Evacuation services	•			•	•
Excavation of new or extension of existing dykes/ditches/droves	•		•		

Finance	•	•	•	•	•
Firebreak and firefighting equipment (where related to wildfires)				•	
Firebreak operations				•	
Flood Protection for Power Stations etc	•				
Forestry Services (incl. Afforestation)	•	•	•	•	•
Gabion installation					•
General supercooling equipment (multi-sector, includes vehicles, manufacturing equipment etc.)		•			
Green roofing and walls	•	•			
Heat-resistant infrastructure (e.g., roads)		•			
Hydropower Reservoir Stations: increase in dam height	•				
Improved New Species	•	•	•	•	
Improvements in Agricultural Management (incl. crop & soil and training)	•	•	•	•	
Improvements in Forest Management	•	•	•	•	
Increase Robustness of Transmission Grids	•	•		•	
Installation of air management systems		•			
Installation of green infrastructure e.g., reed beds, river re-wiggling etc.	•				
Installation of porous hard standing	•				
Installation of temporary flood barriers	•				
Installation of thermal and UV reflective surfaces to buildings		•			

Insurance	•	•	•	•	•
Management of External Mineral and Energy Resources	•	•	•	•	•
Management of Timber Resources	•	•	•	•	•
New Distribution Systems	•	•	•	•	•
New Transmission Systems	•	•	•	•	•
Pest Suppression Systems and Practices	•	•	•	•	•
Pumping equipment	•				
Retrofit building services	•	•			•
Sand bags	•				•
Sinkhole engineering services					•
Smokejumper				•	
Solar cooling (PV powered) to cope with energy demand peaks (only when associated with heatwaves)		•			
Specialist insulative installation		•			
Storm Water (management, treatment, planning)	•				
Stormwater civil engineering for new developments	•				
Stormwater mitigation measures	•				
Structural Surveys	•	•			•
Supply Chain Management (all)	•	•	•	•	•
Supply Chain Management (not mineral or timber)	•	•	•	•	•

Sustainable drainage and water management	•		•		
Sustainable Sourcing Consulting	•	•	•	•	•
Training (Wildfire)				•	
Tree surgery where storm-related					•
Underground Cable Installation	•	•	•	•	•
Underpinning services for landslide mitigation					•
Vegetation management for land stabilization					•
Wastewater management and processing of sewage (drought-relevant activities only)			•		
Water Irrigation			•		
Water use reduction technologies			•		

Supplementary Table 6: **Excerpt of the Taxonomy for the Netherlands in 2018** (purchases by NACE 2 sector in million EUR).

Year	Sectors Purchasing (Total Country Purchase in €m)	Hazard Type	Netherlands
2018	A. Agriculture, Forestry and Fishing	Flooding	25.18
2018	A. Agriculture, Forestry and Fishing	Heatwave	31.61
2018	A. Agriculture, Forestry and Fishing	Drought	3.43
2018	A. Agriculture, Forestry and Fishing	Wildfires	1.38
2018	A. Agriculture, Forestry and Fishing	Other	4.08
2018	B. Mining and Quarrying	Flooding	21.03
2018	B. Mining and Quarrying	Heatwave	32.90
2018	B. Mining and Quarrying	Drought	0.00
2018	B. Mining and Quarrying	Wildfires	1.00
2018	B. Mining and Quarrying	Other	5.31
2018	C. Manufacturing	Flooding	22.69
2018	C. Manufacturing	Heatwave	29.66
2018	C. Manufacturing	Drought	4.74
2018	C. Manufacturing	Wildfires	1.96
2018	C. Manufacturing	Other	3.81
Etc.	Etc.	Etc.	Etc.

Supplementary Note 3: Results – Growth Rates in Adaptation Spending

Supplementary Table 7: Yearly growth rates (in %) in adaptation spending per economic sector over the 28 countries, aggregated over the 5 hazard categories.

Economic Sectors	2019	2020	2021	2022
A. Agriculture, Forestry and Fishing	35.02	36.20	33.64	31.49
B. Mining and Quarrying	37.07	38.88	34.37	30.82
C. Manufacturing	38.45	37.30	35.17	30.80
D. Electricity, Gas, Steam and Air Conditioning Supply	38.09	37.63	35.03	31.07
E. Water Supply; Sewerage, Waste Management and Remediation Activities	36.34	37.76	36.84	28.45
F. Construction	37.64	37.94	36.23	30.57
G. Wholesale and Retail Trade; Repair of Motor Vehicles and Motorcycles	37.98	36.93	36.43	30.16
H. Transportation and Storage	37.38	37.94	35.78	30.89
I. Accommodation and Food Service Activities	38.00	38.51	36.21	29.44
J. Information and Communication	37.58	37.48	35.49	30.84
K. Financial and Insurance Activities	37.03	38.60	35.22	30.49
L. Real Estate Activities	36.93	37.21	36.51	30.92
M. Professional, Scientific and Technical Activities	37.65	37.92	36.12	29.79
N. Administrative and Support Service Activities	36.65	39.45	34.96	30.73
O. Public administration and defence; compulsory social security	36.64	38.06	35.72	30.23
P. Education	38.03	38.57	35.34	31.44
Q. Human Health and Social Work Activities	37.36	39.09	36.75	29.95
R. Arts, Entertainment and Recreation	39.94	37.17	32.94	32.55
S. Other Service Activities	36.59	38.53	35.40	30.85
Average	37.39	37.96	35.48	30.60

Supplementary Table 8*:* Yearly growth rates (in %) in adaptation spending per country over the 19 economic sectors, aggregated over the 5 hazard categories.

**Country**	**2019**	**2020**	**2021**	**2022**
Austria	37.88	37.54	35.80	32.87
Belgium	37.21	37.57	36.84	32.71
Bulgaria	35.13	33.10	31.81	19.62
Croatia	39.09	40.92	36.80	29.63
Cyprus	38.98	40.84	36.55	32.97
Czechia	34.73	39.47	31.23	22.96
Denmark	37.70	36.68	35.23	31.57
Estonia	34.88	39.94	33.55	19.85
Finland	37.85	37.50	37.55	31.94
France	39.61	34.47	38.25	37.07
Germany	38.06	36.67	35.32	34.47
Greece	38.83	40.51	37.89	30.09
Hungary	36.35	42.41	33.33	35.06
Ireland	35.66	42.55	40.31	29.33
Italy	39.86	38.57	37.74	35.17
Latvia	32.07	38.51	34.00	25.91
Lithuania	38.93	36.54	31.53	20.19
Luxembourg	38.28	35.07	32.78	33.18
Malta	36.64	36.20	37.96	31.72
Netherlands	37.26	37.60	35.61	33.49
Poland	38.47	38.82	34.14	30.34
Portugal	38.99	35.57	38.86	32.66
Romania	33.29	38.62	34.32	25.26
Slovakia	38.57	36.99	36.59	32.18
Slovenia	35.79	40.07	37.03	31.03
Spain	37.05	39.58	35.94	33.72
Sweden	42.61	36.12	31.55	39.83
United Kingdom	37.07	34.33	34.97	32.13
Average	37.39	37.96	35.48	30.60

Supplementary Note 4: Methods – Regression Sensitivity Analysis

Below, we present the sensitivity analysis regarding hazard and temporal differences discussed in the **Methods**, and compare it to the main regression presented in the **Results**. We complement this with a jackknife analysis in which the main regression is conducted after removing each sector, country and hazard type individually. The purpose of this methodology is to discern if belonging to a specific group (country, sector, hazard type) has a disproportionate impact on the regression estimates. Due to the length of the table, the jackknife analysis can be found in our Zenodo repository: https://doi.org/10.5281/zenodo.14288580

Supplementary Table 9: Regression Sensitivity Analysis of the relationship between public and private adaptation investment growth. Note that D. represents a first difference (which combined with the natural log of a variable approximates a growth rate) and L. represents a one-year lag. Our **Main** regression shows the results of the analysis already presented in the paper. For comparison, we produce hazard specific regressions (**Drought**, **Flooding**, **Heatwave**, **Other**, **Wildfire**) with fewer observations, and a final **Time-Hazard** regression in which we interact hazard and year-specific effects. The negative coefficients for D.lnpublicadapt (i.e., public adaptation spending growth) can be explained by the year-specific effects, which become positive for all hazards in 2022. Note that lnadaptinvest represents the natural log of private adaptation investment.

VARIABLES (D.lnadaptinvest=)	*Main*	*Drought*	*Flooding*	*Heatwave*	*Other*	*Wildfires*	*Time-Hazard*
D.lnpublicadapt	0.279***	0.369***	-0.218***	-0.237***	-0.177***	-0.187***	
	(0.062)	(0.073)	(0.048)	(0.044)	(0.039)	(0.040)	
D.lnpublicadapt(Drought 2019)							-0.107
							(0.127)
D.lnpublicadapt(Drought 2020)							0.190
							(0.124)
D.lnpublicadapt(Drought 2021)							0.603***
							(0.095)
D.lnpublicadapt(Drought 2022)							0.877***
							(0.147)
D.lnpublicadapt(Flooding 2019)							-1.131***
							(0.078)
D.lnpublicadapt(Flooding 2020)							-0.586***
							(0.047)
D.lnpublicadapt(Flooding 2021)							-0.055
							(0.040)
D.lnpublicadapt(Flooding 2022)							0.496***
							(0.063)
D.lnpublicadapt(Heatwave 2019)							-0.935***
							(0.070)
D.lnpublicadapt(Heatwave 2020)							-0.412***
							(0.044)
D.lnpublicadapt(Heatwave 2021)							0.127***
							(0.046)
D.lnpublicadapt(Heatwave 2022)							0.738***
							(0.079)
D.lnpublicadapt(Other 2019)							-1.049***
							(0.073)
D.lnpublicadapt(Other 2020)							-0.519***
							(0.047)
D.lnpublicadapt(Other 2021)							0.037
							(0.041)
D.lnpublicadapt(Other 2022)							0.598***
							(0.069)
D.lnpublicadapt(Wildfires 2019)							-1.150***
							(0.082)
D.lnpublicadapt(Wildfires 2020)							-0.579***

							(0.047)
D.lnpublicadapt(Wildfires 2021)							-0.050
							(0.041)
D.lnpublicadapt(Wildfires 2022)							0.486***
							(0.059)
lnadaptinvest = L,	-0.096***	-0.758***	-0.063***	-0.070***	-0.041***	-0.056***	-0.588***
	(0.009)	(0.073)	(0.009)	(0.009)	(0.006)	(0.008)	(0.037)
lngva = D,	0.030***	0.118	0.008	-0.006	-0.003	0.013	0.007
	(0.007)	(0.288)	(0.010)	(0.010)	(0.008)	(0.010)	(0.005)
deflator = L,	0.010***	0.069***	0.005***	0.005***	0.003***	0.004***	0.012***
	(0.001)	(0.012)	(0.001)	(0.001)	(0.001)	(0.001)	(0.001)
Population Density	0.000**	-0.003	0.000***	0.000**	0.000	0.000**	0.000
	(0.000)	(0.002)	(0.000)	(0.000)	(0.000)	(0.000)	(0.000)
Sovereign Debt Rating	0.043***	0.321***	0.015**	0.025***	0.011**	0.013**	0.040***
	(0.006)	(0.083)	(0.006)	(0.006)	(0.005)	(0.006)	(0.005)
*Flooding*eventcount_5year*	0.003***		0.002**				0.002**
	(0.001)		(0.001)				(0.001)
*Heatwave*eventcount_5year*	0.010***			0.012***			0.007***
	(0.003)			(0.003)			(0.003)
*Other*eventcount_5year*	-0.000				-0.000		0.000
	(0.000)				(0.000)		(0.000)
*Wildfires*eventcount_5year*	0.006***					0.004**	-0.001
	(0.002)					(0.002)	(0.002)
Constant	-1.528***	-11.992***	-0.314*	-0.509***	-0.098	-0.385**	-1.271***
	(0.200)	(2.028)	(0.176)	(0.172)	(0.142)	(0.181)	(0.139)
Observations	8,288	224	2,016	2,016	2,016	2,016	8,288
R-squared	0.169	0.524	0.102	0.113	0.095	0.117	0.425
Fixed effects (Country/Sector/Year)	YES	YES	YES	YES	YES	YES	YES
Clustering	YES	YES	YES	YES	YES	YES	YES

Robust standard errors in parentheses
**** p<0.01, ** p<0.05, * p<0.1*

Supplementary References

1. Howard, S., Howard, S. & Howard, S. Quantitative market analysis of the European Climate Services sector – The application of the kMatrix big data market analytical tool to provide robust market intelligence. *Clim Serv* **17**, 100108 (2020).

2. Jaikumar, R. Postindustrial manufacturing. *Harv Bus Rev* **64**, 69–76 (1986).
